# Protection against Neurological Symptoms by Consuming Corn Silk Water Extract in Artery-Occluded Gerbils with Reducing Oxidative Stress, Inflammation, and Post-Stroke Hyperglycemia through the Gut-Brain Axis

**DOI:** 10.3390/antiox11010168

**Published:** 2022-01-16

**Authors:** Jin Ah Ryuk, Byoung Seob Ko, Na Rang Moon, Sunmin Park

**Affiliations:** 1 Korean Medicine Convergence Research Division, Korea Institute of Oriental Medicine, Daejeon 305-811, Korea; yukjinah@kiom.re.kr (J.A.R.); bsko@kiom.re.kr (B.S.K.); 2 Department of Food & Nutrition, Obesity/Diabetes Center, Hoseo University, Asan 336-795, Korea; a213900@hanmail.net; 3 Department of Bioconvergence System, Hoseo University, Asan 336-795, Korea

**Keywords:** ischemic stroke, proinflammatory cytokines, post-stroke hyperglycemia, gut microbiome

## Abstract

Corn silk (*Stigma maydis*), rich in flavonoids, is traditionally used to treat edema, depression, and hyperglycemia and may alleviate ischemic stroke symptoms in Chinese medicine. This study examined whether corn silk water extract (CSW) could alleviate ischemic stroke symptoms and post-stroke hyperglycemia in Mongolian gerbils with transient cerebral ischemia and reperfusion (I/R). After being given 0.05% (I/R-LCSW) and 0.2% (I/R-HCSW), 0.02% aspirin (I/R-aspirin), and cellulose (I/R-control) in their 40 energy% fat diets for three weeks, the gerbils underwent an artery occlusion for eight minutes and reperfusion. They took the assigned diet for an additional three weeks. Sham-operated gerbils without artery occlusion had the same diet as Sham-control. CSW intake reduced neuronal cell death in gerbils with I/R and dose-dependently improved the neurological symptoms, including drooped eyes, crouched posture, flexor reflex, and walking patterns. CSW intake also alleviated the short-term memory and spontaneous alteration and grip strength compared to the I/R-control group. The protection against ischemic stroke symptoms was associated with the reduced tumor necrosis factor-α, interleukin-1β, superoxide, and lipid peroxide levels, promoting superoxide dismutase activity in the hippocampus in the CSW groups, compared to the I/R-control. The blood flow measured by Doppler was improved with CSW compared to the I/R-control. Furthermore, CSW intake prevented the post-stroke hyperglycemia related to decreasing pancreatic β-cell mass as much as the Sham-control, and it was related to protection against β-cell apoptosis, restoring the β-cell mass similar to the Sham-control. CSW intake elevated the relative abundance of *Lactobacillus*, *Bifidobacterium*, *Allobaculum*, and *Akkermansia* compared to the I/R-control. Picrust2 analysis showed that CSW increased the propionate and butyrate metabolism and the starch and glucose metabolism but reduced lipopolysaccharide biosynthesis compared to the I/R-control. In conclusion, CSW intake protects against neuronal cell death and post-hyperglycemia by reducing oxidative stress and inflammation and increasing blood flow and the β-cell mass. The alleviation was associated with promoting the gut-brain axis by changing the gut microbiome community.

## 1. Introduction

A stroke is local neuronal cell death in the brain, in which blood flow is blocked or reduced by a blockage or bleeding in the cerebral vessels, which are called a cerebral stroke and hemorrhagic stroke, respectively [1]. Globally, stroke had the second-highest mortality (11% of the total) from total deaths in 2015 [2]. Ischemic stroke has increased over the last two to three decades, and it is globally higher than hemorrhagic stroke. A stroke induces abnormal movement, dizziness, vision impairment, or speaking loss. A stroke event can reoccur once it is induced. If symptoms disappear in less than one or two hours, it is called a transient ischemic attack. Otherwise, the symptoms last permanently and result in long-term disability [1].

Primary risk factors for stroke include elevated blood pressure and blood glucose, dyslipidemia, and obesity. Lifestyle factors linked to stroke include alcoholism, a high-fat diet, and a sedentary lifestyle. The combined effects of the risk factors can synergistically increase the risk of stroke, and hyperglycemia during the post-stoke period worsens the long-term prognosis. [3,4,5]. Previous studies showed that peroxisome proliferator-activated receptor (PPAR)-γ, insulin sensitizers, reduced recurrent stroke to 66% (95% CI = 44–99%) in patients with stroke or transient ischemic attack compared to the placebo in a meta-analysis of three randomized clinical trials (RCT) [6]. Stroke also affects the gut microbiome, and managing the gut microbiota may be an essential strategy in stroke intervention. It has been an area of recent interest in studying the etiology, progression, and recovery from stroke: the bidirectional activity of the brain and gut that is often referred to as the gut-brain axis [7].

Ischemic stroke events induce oxidative stress and inflammation that play a critical role in the etiology of ischemic brain injury. An antioxidant (ebselen) and γ-polyglutamic acid (γ-PGA)-rich chungkookjang (short-term fermented soybeans with *Bacillus amyloliquefaciens*) alleviated the ischemic stroke symptoms and post-stroke hyperglycemia in ischemia-induced gerbils [4,5]. Herbal medications that exert antioxidant and anti-inflammatory activities also protect against total and ischemic stroke [8]. Dietary antioxidant intake from various foods and beverages is inversely associated with ischemic stroke in women [9]. Therefore, some herbs containing polyphenols and flavonoids may protect against ischemic stroke and alleviate its outcomes. Corn silk (*Stigma maydis*) is rich in flavonoids, such as luteolin 7-*O*-neohesperidoside, 2″-*O*-α-L-rhamnosyl-6-C-fucosyl-3′-methoxyluteolin, 2″-*O*-α-L-rhamnosyl-6-C-quinovosylluteolin, 2″-*O*-α-L-rhamnosyl-6-C-fucosylluteolin, maysin-glycoside, and 3′-methoxymaysin, and terpenoids, such as cis-α-terpinol, citronellol, 6,11-oxidoacor-4-ene, trans-pinocamphone, eugenol, neo-iso-3-thujanol, and cis-sabinene hydrate [10]. These compounds in corn silk are responsible for antioxidant and anti-inflammatory activities. Parent flavonoid bioavailability is often relatively low, but its secondary metabolites have demonstrated various functionalities to alleviate cognitive dysfunction, platelet aggregation, insulin resistance, and cardiovascular diseases [11,12,13]. Furthermore, the functionalities of polyphenols, including flavonoids, are related to the gut microbiota community, of which changes are indirectly involved in neuronal cell survival through the gut-brain axis [13].

Corn silk has been used traditionally for treating edema, hyperglycemia, depression, and fatigue [10]. Meta-analysis with RCT studies (*n* = 567) has shown that corn silk tea supplementation with anti-hypertensive medication effectively reduces blood pressure compared to an anti-hypertensive drug alone, but these RCT studies had poor quality control [14]. Another meta-analysis has reported that corn silk tea has the potential to alleviate dyslipidemia in patients with angina pectoris [15]. Therefore, corn silk could be an effective intervention for the prevention and/or treatment of recurrent ischemic stroke. These effects may be due to improved blood pressure and blood glucose management. However, ischemic stroke may also be related to the gut microbiota community, but the anti-stroke effects of corn silk have not yet been investigated. Nevertheless, the anti-oxidative and anti-inflammatory properties of corn silk polyphenols and flavonoids may protect against neuronal cell death by ischemic stroke and also modulate the gut microbiota. The effects of corn silk extracts were examined in an animal model of ischemic stroke. Mongolian gerbils are suitable for studying ischemic stroke since they are prone to inducing cerebral ischemia by the transient occlusion of the two carotid arteries, showing the general features of human stroke [7]. Therefore, this study aimed to examine the effects of corn silk on ischemic stroke outcomes and post-stroke hyperglycemia in male Mongolian gerbils fed a corn silk water extract. Its mechanism was also explored in the aspects of oxidative stress, inflammation, and gut microbiota.

## 2. Materials and Methods

### 2.1. Water Extract Preparation of Corn Silk

Corn silk was washed and cut into small pieces with a hay cutter. They were extracted with distilled water (1:10, *w*/*v*) at 90 °C for 2 h and filtered through 50 μm filter paper. The filtrates were concentrated at 60 °C under vacuum conditions, and the concentrates were lyophilized. The yield was 12.4%. The total phenol contents expressed as mg gallic acid equivalents g^−1^ were determined using the Folin–Ciocalteu method [16]. The total flavonoid contents were measured using the modified method reported by Davis [17] and were calculated as mg rutin equivalents g^−1^.

### 2.2. Animals and Diets

Seven-week-old male Mongolian gerbils (*Meriones unguiculatus*) were purchased from DaehanBio (Eumsung, Korea) and acclimated in the animal facility for one week. They were raised in an individual cage at 23 °C, 60% humidity, and a 12 h light/dark cycle. They freely consumed food and water. The study protocols conformed to the National Institute of Health Guidelines and were approved by Hoseo University Animal Care and Use Committee (HSIAUC-18-065).

A semi-purified diet formulation for laboratory rodents as formulated by the American Institute of Nutrition in 1993 (AIN-93) was used to make an experimental diet [18]. The fat contents in AIN-93 were modified to 43 energy percent, and corn silk water extract (0.05% and 0.2%) was added to the 43 energy percent (En%) fat diet. For the I/R-aspirin, 0.02% aspirin (Bayer, Leverkusen, Germany) was added to the 43 En% fat diet as the positive control (I/R-aspirin). The corn silk water extracts and aspirin were substituted for the cellulose contents. According to the analysis results, each diet was tailored to contain identical total amounts of carbohydrates, protein, and lipids by changing the casein, soybean oil, and cellulose contents. All diets consisted of 43 En% carbohydrates, 17 En% protein, and 40 En% fats. Cellulose (3.4%), mineral (3.5%), and vitamin (1.0%) mixtures were added to all diets.

### 2.3. Induction of Transient Forebrain Ischemia and Reperfusion (I/R) in Gerbils

Intraperitoneal injections of 400 mg/kg of chloral hydrate (Sigma, St. Louis, MO, USA) were used for anesthesia prior to surgery. Occlusion and reperfusion of common arteries were used to generate the ischemic stroke model. It was accomplished by gaining access to and clamping the common arteries with aneurysm clips through an incision in the neck. After 8 min the clips were removed to enable reperfusion [4,19]. An ophthalmoscope (Hill-Rom, Chicago, IL, USA) was used to observe the blockage and restoration of blood flow in the eyes. Body temperature was maintained at 37 ± 0.5 °C and monitored using a rectal temperature probe (TR-100; Fine Science Tools, Foster City, CA, USA) during the I/R surgery to minimize the potential for cerebral ischemia due to low body temperature [19]. Following the procedure, the wound was closed with a 3.0 silk suture, and the gerbils remained in a thermal incubator (Mirae Medical Industry, Seoul, Korea) to maintain their body temperature for 12 h. Gerbils underwent a sham operation of I/R without the occlusion of the common carotid arteries. After the surgery, all animals were included, and there were no exclusion criteria.

### 2.4. Experimental Design and Metabolic Analysis

The ischemic-stroke-induced gerbils were divided randomly into four groups by dice, and each group was randomly allocated into the control or treatment groups. The assigned four groups were as follows: (1) I/R + 43 En% fat diet (I/R-control), (2) I/R + 0.05% corn-silk-water-extract-added diet (I/R + LCSW), (3) I/R + 0.2% corn silk water extract (I/R-HCSW), and (4) I/R + 0.02% aspirin-added diet (I/R-aspirin). Gerbils with the sham operations had a 43 En% fat diet as the Sham-control group. Each group included ten gerbils calculated from the G-Power program based on an effect size using stroke symptoms = 0.4, α = 0.05, and power = 0.8. Rats were randomly allocated into each group. 

**Figure 1:**
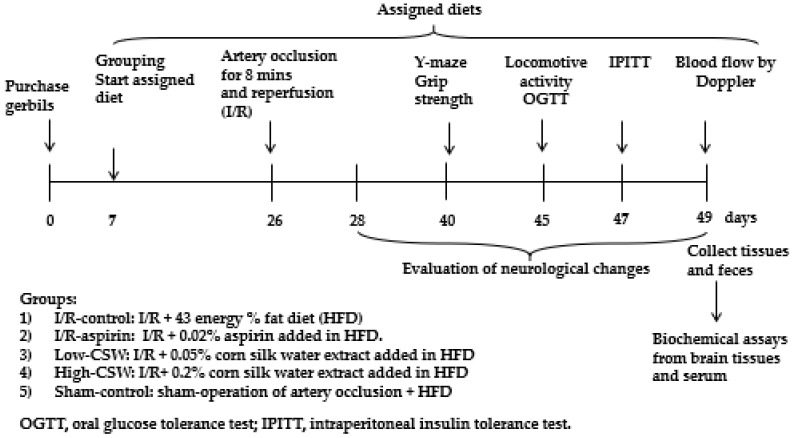
Experimental design.

Figure 1 outlines the study design. Food consumption, body weight, and serum glucose concentrations were measured weekly. On the 27th day post-ischemia, an oral glucose tolerance test (OGTT) was conducted by orally providing 2 g glucose/kg body weight and measuring serum glucose and insulin concentrations for 120 min [20]. On the following day, 6 h fasted animals were subjected to an intraperitoneal insulin tolerance test (IPITT) by the intraperitoneal injection of 0.75 IU/kg body weight and measured serum glucose concentrations every 15 mins [20]. Blood flow was measured two days after the IPITT after 16 h fasting. Serum glucose and insulin levels were measured using a Glucose Analyzer II (Beckman, Palo Alto, CA, USA) and an ultrasensitive rat mouse insulin kit (Crystal Chem, Elk Grove Village, IL, USA), respectively.

### 2.5. Neurological Severity Score and Grip Strength

The artery occlusion outcomes were scored with a neurological evaluation as follows: eyelid drooped (no symptom = 0, one eyelid partially drooped = 1, one eyelid totally drooped = 2, both eyelids partially drooped = 3, both eyelids totally drooped = 4); hair bristling (no symptom = 0, hair bristled = 1); decreased muscular tone (no symptom = 0, diminished muscular tone or strength in the limbs = 1); flexor reflex after pinching hind limbs(no symptom = 0, hind limbs slightly withdrawn = 1, no withdrawal = 2); posture (normal = 0, hunched = 1); and walking style (normal = 0, slow = 1, none = 2).

The grip strength of the forelimb was assessed using a Grip Strength Meter (GPM-100; Melquest, Toyama, Japan). Briefly, after a gerbil grasped the bar mounted on a force gauge and its reading was stabilized, the trained researcher slowly pulled back the tail of the gerbil. The peak pull force was measured in grams [21].

### 2.6. Memory Impairment Assessment Using Y Maze Tests

A horizontal Y-shaped maze, constructed with three arms of 50.5 cm in length, 20 cm in width, and 20 cm in height, was used to measure the short-term memory. A gerbil was placed in one arm, and its movements were monitored for 8 min. The number of correct consecutive entries into each arm of the Y maze was measured [22]. The percentage of a right consecutive alternation among the total entries into the three arms was calculated, and a higher percentage indicated a better short-term memory.

### 2.7. Blood Flow Determination

A heating pad was used to maintain a constant body temperature of 37.0 ± 0.2 °C using during the final surgical procedures. Gerbils were anesthetized with ketamine and xylazine, one of the carotid arteries surgically exposed, and a Doppler flow probe (TSD145, BIOPAC Systems, Inc., Goleta, CA, USA) was placed under the exposed artery. After 3 min, platelet aggregation was initiated by a 2 min topical application of 20% FeCl_3_-saturated filter paper (1.5 × 1.5 mm) and then washing the common carotid artery with saline. Arterial blood flow was continuously monitored for 15 min by Laser Doppler Flowmetry (LDF100C-1, BIOPAC Systems, Inc., Goleta, CA, USA) [23]. Total arterial occlusion was defined as a complete lack of blood flow in the carotid artery and was expressed in blood perfusion units (BPU), a relative unit scale of vascular blood flow determined by the degree of unclogging of the platelet aggregation in the blood. Peak BPU and time to return to normal BPU were measured.

After finishing blood flow measurement, blood, organs, and feces from the cecum were collected under anesthesia with ketamine and xylazine, and serum was separated after centrifugation at 3000× *g* at 4 °C for 10 min. Serum and organs were stored at −70 °C until further analysis. Serum tumor necrosis factor-α (TNF-α) and interleukin (IL)-1β concentrations were determined using ELISA kits (R & D Systems, Minneapolis, MN, USA).

### 2.8. Determination of Lipid Peroxidation, Superoxide Anion Contents, and Superoxide Dismutase Activity in the Hippocampus

The hippocampus was homogenized in lysis buffer (pH 7.2), and the supernatants were separated after centrifuging at 1580× *g* for 15 min at 4 °C. As an indicator of lipid peroxidation, hippocampal malondialdehyde (MDA) contents were measured. MDA formed a colored complex in the presence of 2-thiobarbituric acid, which was detected at 532 nm using a spectrophotometer (Perkin Elmer). 1,1′,3,3′-Tetraethoxypropane was used as the standard, and the MDA contents in the hippocampus tissue were expressed as μmol/g protein.

The superoxide anion contents in the supernatants were measured with the reaction with nitro blue tetrazolium. The end product was a colored formazan product formed from blue tetrazolium quantified at 560 nm using a spectrophotometer (Perkin Elmer, Waltham, MA, USA) [24]. The superoxide dismutase (SOD) activity was measured in the supernatants that reacted with a highly water-soluble tetrazolium salt (WST) and enzyme working solution to measure SOD activity [24].

### 2.9. Real-Time Quantitative Reverse Transcriptase-Polymerase Chain Reaction (RT-PCR) in the Hippocampus and Liver

Total RNA was isolated from the hippocampus and liver using Trizol reagent (Gibco-BRL, Rockville, MD, USA), and the cDNA was synthesized from total RNA using polymerase chain reaction (PCR) [25]. The genes of interest, including hippocampal IL-1β, TNF-α, and β-actin and hepatic fatty acid synthase (FAS) and sterol regulatory element-binding protein-1c (SREBP1c), were quantified with the respective primers using Sybergreen mix by a real-time PCR machine (BioRad Laboratories, Hercules, CA, USA) [4]. The gene expression levels in the islets were quantitated using the comparative 2^−ΔΔCT^ method [26].

### 2.10. Cresyl Violet Staining

The neuronal cell death resulting from the artery occlusion was evaluated with cresyl violet remaining in the hippocampal sections from the gerbils at the end of the 28-day experimental period. The brain tissues were soaked overnight in 30% sucrose, and the solution was then removed. The frozen brain was sectioned serially on a cryostat (Leica, Wetzlar, Germany) into 30 µm coronal sections collected in six-well plates containing phosphate-buffered saline. The sections were mounted on gelatin-coated microscopy slides. Cresyl violet acetate (1.0% *w*/*v*, Sigma, St. Loise, MO, USA) was dissolved in a 0.6% glacial acetic acid solution (Sigma, St. Loise, MO, USA) to make a concentration of 0.1%. After staining the brain sections with a cresyl violet solution for 2 min at room temperature, they were washed twice in distilled water. The fixed brain tissues were dehydrated by immersion in a graded series of ethanol solutions at room temperature and mounted with Permount (Fisher Scientific Inc., Pittsburgh, PA, USA).

### 2.11. Islet Morphometry by Immunohistochemistry

After the experimental intervention, four gerbils from each group were selected for treatment with 5-Bromo-2-deoxyuridine (BrdU; Roche Molecular Biochemicals; 100 µg/kg body weight). The pancreas of each gerbil was surgically removed at 6 hours post-injection, fixed overnight in 4% paraformaldehyde (pH 7.2, ambient temperature), and embedded in paraffin blocks. Serial 5 μm tissue sections were prepared and mounted on slides. After rehydration, every sixth or seventh pancreas section was selected to determine the β-cell area, BrdU incorporation, and apoptosis, taking care to avoid repeated use of pancreatic sections having the same islet information.

Endocrine β-cells were stained by incubating the selected paraffin-embedded pancreatic sections with a guinea pig anti-insulin antibody. As a percent of the total pancreatic area, the anti-insulin antibody-stained areas were used to determine β-cell proliferation as identified by BrdU incorporation int β-cells of the gerbils. This incorporation was determined by immunohistochemistry with anti-insulin (Zymed Laboratories, South San Francisco, CA, USA) and anti-BrdU antibodies (Roche, Mannheim, Germany). Apoptosis of the β-cells was measured using a TUNEL assay (Roche, Basel, Switzerland) with hematoxylin and eosin [7]. The number of the BrdU^+^ cells and apoptotic bodies in the islets was calculated based on the total islet numbers [7].

### 2.12. Measurement of the Serum Short-Chain Fatty Acid (SCFA) Concentrations

The serum was mixed with the solvent containing n-butanol, tetrahydrofuran, and acetonitrile (Duksan, Ansan-Si, Korea), and HCl was added to the mixture. The mixture was vortexed, and the supernatant separated after centrifuging at 15,000× *g* for 5 min on 4 °C. The SCFA in the supernatants was measured by gas chromatography (GC Clarus 680 GAS, PerkinElmer, Waltham, MA, USA) using an Elite-FFAP 30 m × 0.25 mm × 0.25 μm capillary column, with helium as the carrier gas at a flow rate of 1 mL/min.

### 2.13. Next-Generation Sequencing (NGS) of Gut Microbiomes

The gut microbiome composition was measured in the feces by metagenome sequencing using NGS. Bacterial DNA was extracted from the fecal samples using a Power Water DNA Isolation Kit (MoBio, Carlsbad, CA, USA). Each library was prepared using the PCR product, as described by the GS FLX plus Library Preparation Guide. The emPCR corresponding to the purified library’s clonal amplification was carried out using the GS-FLX plus emPCR Kit (454 Life Sciences, Branford, CT, USA) [27]. Libraries were immobilized on DNA capture beads. The beads were added to the amplification mix and oil and shaken vigorously on a Tissue Lyser II (Qiagen, Valencia, CA, USA) to form the micro-reactors containing the amplification mix and a single bead. Emulsions of the micro-reactor mix were dispensed into a 96-well plate. The PCR amplification program was run with 16S universal primers in the FastStart High Fidelity PCR System (Roche, Basel, Switzerland) [27,28]. The sequencing of bacterial DNA in the feces was performed by Macrogen Inc. (Seoul, Korea) using a Genome Sequencer FLX plus 454 (Roche, Basel, Switzerland).

Mothur v.1.36 was used to analyze the 16S amplicon sequencing [20]. The Miseq standard operating protocol was used to identify and enumerate the bacteria in all fecal samples. The sequences were aligned using Silva reference alignment v.12350, and the sequences found to be mitochondrial, Eukaryota, or unknown were removed. The operational taxonomic units (OTUs) delimited at 98% identity and taxonomically classified by consensus using Silva reference were selected. A relaxed neighbor-joining tree with one representative sequence per OTU was obtained using Clearcut after calculating the uncorrected pairwise distances between the aligned reads. The α-diversity was calculated with Shannon and Chao index, and bacteria counts of each taxonomy were assessed. The β-diversity was determined with the unweighted UniFrac distance method. Principal coordinates analysis (PCoA) was conducted using the R package with the OTU-abundance table converted to the relative abundance.

### 2.14. Gut Microbiota Metabolism Predicted by PICRUSt2 Pipeline Analysis

Microbial metabolism in the gut was predicted by NGS results using PICRUSt2. The biome file was used to generate the final FASTA and count table files by the Mothur program. Metabolic pathways of the microbiota were predicted from picrust2_pipeline.py (https://github.com/picrust/picrust2/wiki/Full-pipeline-script, accessed on 7 May 2020). An assortment of metabolic profiles as described in the Kyoto Encyclopedia of Genes and Genomes (KEGG) Orthologues (KO) were identified using the KEGG mapper (https://www.genome.jp/kegg/tool/map_pathway1.html, accessed on 17 May 2020) [29].

### 2.15. Statistical Analysis

All results are expressed as the means ± standard deviation (SD). Statistical analysis was performed using SAS software. The significance of the treatment effects on neurological impairment and glucose homeostasis after cerebral ischemia was determined by one-way ANOVA followed by a Tukey’s test. The comparison between the I/R-control and Sham-control groups was tested using a two-sample *t*-test. Differences among the groups with a *p* < 0.05 were considered significant.

## 3. Results

### 3.1. Total Phenols and Flavonoids in the Corn Silk Water Extract

The total phenol and flavonoid contents were 183.2 ± 1.5 and 110.4 ± 0.02 mg/g dried water extract of corn silk, respectively.

### 3.2. Energy Metabolism

The body weight gain was lower during the first three weeks before the artery occlusion in the I/R-aspirin and CSW groups. The CSW exhibited lower body weight gain than the I/R-aspirin group. After the occlusion, however, the body weight gain during two weeks was much lower in the I/R-control group than the I/R-aspirin group. The I/R-aspirin and I/R-HCSW groups inhibited the decrease (Table 1). During the first three weeks, food intake was lower in the I/R-aspirin and CSW groups. On the other hand, food intake was similar among the groups after artery occlusion (Table 1).

### 3.3. Neuronal Cell Death and Memory-Related Brain Function

**Figure 2:**
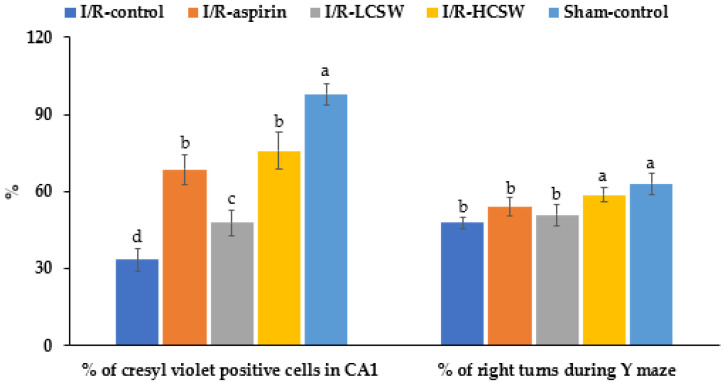
Brain cell death and memory loss after ischemic stroke. Gerbils that underwent the transient forebrain ischemia by carotid artery occlusion for 8 min and reperfusion were randomly divided into four groups, as follows: (1) 0.2% cellulose (I/R-control), (2) 0.02% aspirin (I/R-aspirin), (3) 0.05% freeze-dried corn silk water extract (I/R-LCSW), and (4) 0.2% CSW (I/R-HCSW) in a high-fat diet. Sham-operated gerbils without artery occlusion had the same diet as Sham-control. At the end of the experiment, the percentage of cresyl-violet-positive cells in the hippocampal CA1 region was quantified by densitometry in the cresyl violet staining. Dots and bars represent means ± SD (*n* = 10). ^a–d^ Different letters on the bars indicate significant differences in the means of the designated groups by Tukey’s test at *p* < 0.05.

Blue staining indicating live hippocampal neuronal cells was much lower in the I/R-control than the Sham-control. The I/R-LCSW and I/R-HCSW groups showed a dose-dependent inhibition of the decrease in blue staining in the brain section (*p* < 0.05; Supplemental Figure S1). The CSW-fed gerbils dose-dependently showed higher neuronal cell survival after artery occlusion (Figure 2).

Short-term memory impairment in the artery-occluded gerbils was measured using the Y maze (Figure 2). The percentage of correct alteration during the Y maze test was lower in the I/R-control group than the Sham-control group, while the CSW groups improved the short-term memory deficit in a dose-dependent manner (*p* < 0.05; Figure 2). The results indicated that CSW alleviated short-term memory impairment in artery-occluded gerbils.

### 3.4. Assessment of Neurological Symptoms

**Figure 3:**
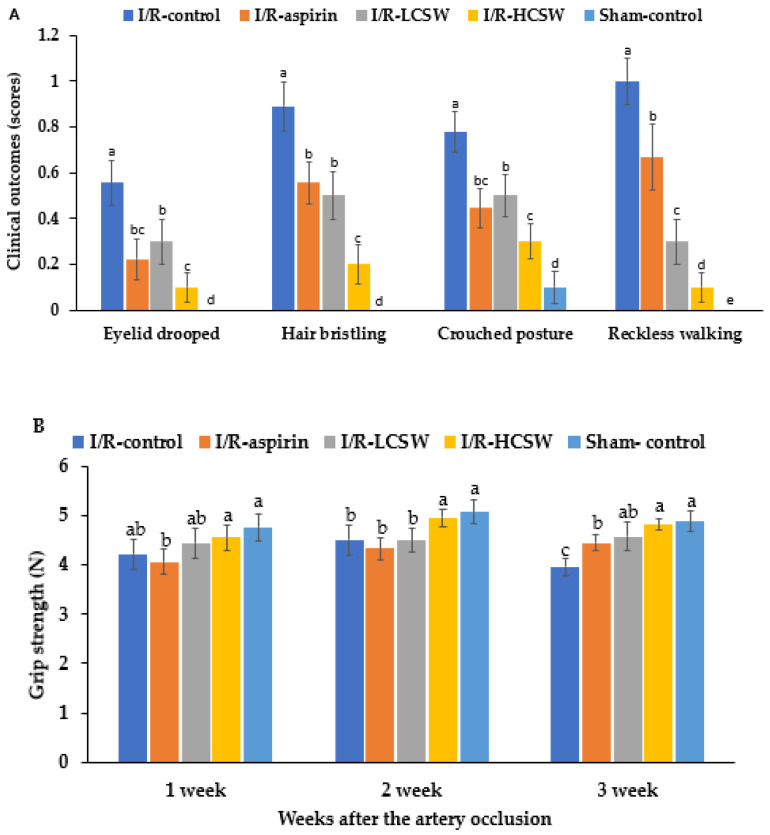
Neurological severity scores. Gerbils that underwent the transient ischemic attack by carotid artery occlusion for 8 min and reperfusion were randomly divided into four groups, as follows: (1) 0.2% cellulose (I/R-control), (2) 0.02% aspirin (I/R-aspirin), (3) 0.05% freeze-dried corn silk water extract (I/R-LCSW), and (4) 0.2% CSW (I/R-HCSW) in a high-fat diet. Sham-operated gerbils without artery occlusion had the same diet as Sham-control. The neurological symptoms, including drooping eyelid, crouched posture, and hair bristling (**A**); flexor reflex, walking patterns, and force to grip the bar (**B**), were shown at the third week after inducing ischemic stroke attack. Bars represent means ± SD (*n* = 10). ^a–e^ Different letters on the bars indicate significant differences in the means of the designated groups by Tukey’s test at *p* < 0.05.

After inducing an artery occlusion, the neurological symptoms, including drooping eyelid, hair bristling, crouched posture, and reckless walking, improved over time in all gerbils given the artery occlusion (Figure 3). The scores of the clinical neurological symptoms at the third week were higher in the I/R-control group than the Sham-control, while the CSW and I/R-aspirin groups showed a decrease. The I/R-HCSW group exhibited the lowest scores for the clinical neurological symptoms among the groups of artery-occluded gerbils (*p* < 0.05; Figure 3A), although they did not recover the clinical symptoms completely. The grip strength was lower in the I/R-control group than the Sham-control group after three weeks of the artery occlusion. In the third week, the grip strength was much lower in the control group than in the other groups. The grip strength increased in the I/R-HCSW similar to the Sham-control (*p* < 0.05; Figure 3B).

### 3.5. Blood Flow and Lipid Profiles

Peak BPU was lower in controls than in the Sham-control group but differed between I/R-control and the I/R-aspirin. In contrast, CSW dose-dependently increased peak BPU (Table 2). Clot removal time was longer in the I/R-control than Sham-controls but was shorter in I/R-LCSW and I/R-HCSW. The removal time in the I/R-HCSW group was equivalent to the I/R-aspirin group; aspirin is a well-established anti-thrombotic agent (*p* < 0.05; Table 2). The Doppler test results suggest that CSW may have prevented occlusion-induced brain cell death, possibly, via improving blood flow by preventing or dissolving blood clots. 

Serum concentrations of total and LDL cholesterol as well as triacylglycerols, but not HDL-cholesterol, were elevated in the I/R-control group (Table 2). The changes in circulating lipids also affected blood flow. I/R-HCSW normalized LDL and triglyceride concentrations compared to the I/R-control, but serum triacylglycerols were only lowered in the I/R-aspirin group (Table 2).

### 3.6. Glucose Metabolism

The serum glucose concentrations at the fasting state were higher in the I/R-control group than in the Sham-control group, while the serum insulin concentrations were opposite to the serum glucose concentrations. The I/R-LCSW inhibited increased fasting serum glucose concentrations as much as the I/R-aspirin, whereas the I/R-HCSW lowered them similar to the Sham-control (Table 2). The I/R-HCSW, but not the I/R-LCSW, increased the serum insulin concentrations similar to the I/R-aspirin and Sham-control (*p* < 0.05; Table 2). Thus, the increase in serum glucose concentrations was related to the decreased serum insulin concentrations and insulin sensitivity after the artery occlusion; the CSW and I/R-aspirin groups showed a smaller decrease.

**Figure 4:**
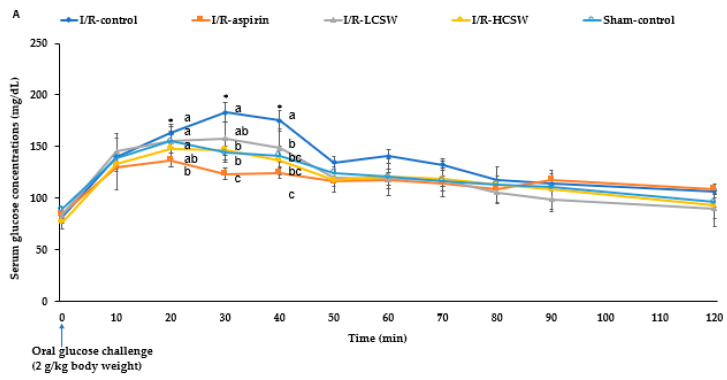

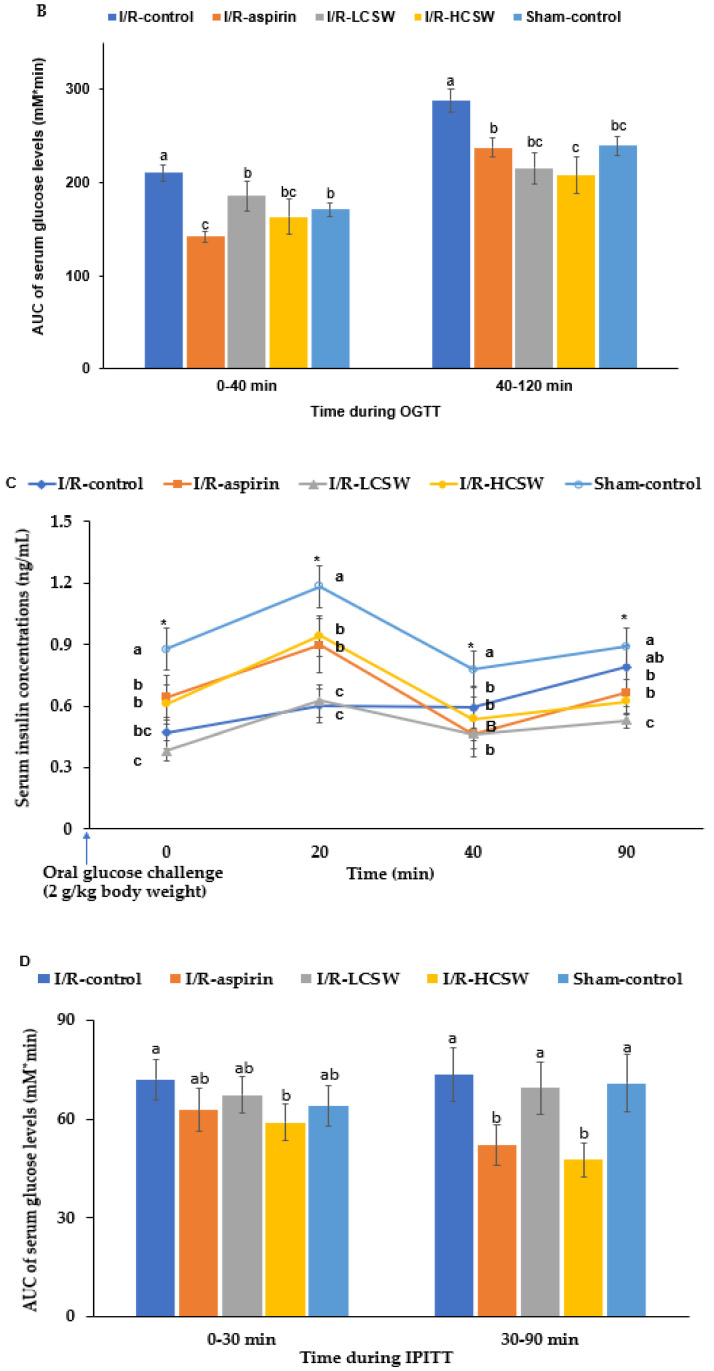
Oral glucose tolerance test (OGTT) and intraperitoneal insulin tolerance test (IPITT). Gerbils that underwent the transient forebrain ischemia by carotid artery occlusion for 8 min and reperfusion were randomly divided into four groups, as follows: (1) 0.2% cellulose (I/R-control), (2) 0.02% aspirin (I/R-aspirin), (3) 0.05% freeze-dried corn silk water extract (I/R-LCSW), and (4) 0.2% CSW (I/R-HCSW) in a high-fat diet. Sham-operated gerbils without artery occlusion had the same diet as Sham-control. After overnight fasting, gerbils had an OGTT with 2 g glucose/kg body weight. The changes of serum glucose levels during 120 min (**A**) and area under the curve (AUC) of serum glucose concentrations (**B**) in the first part (0–40 min) and the second part (40–120 min) during OGTT. Serum insulin concentrations were also measured during OGTT (**C**). The next day gerbils underwent an IPITT with 1 IU insulin/kg body weight after 6 h food deprivation. The area under the curve (AUC) of serum glucose in the first part (0–30 min) and the second part (30–90 min) during the IPITT (**D**). Bars and dots represent means ± SD (*n* = 10). * Significantly different among the groups at *p* < 0.05. ^a–c^ Different letters on the bars indicate significant differences in the means of the designated groups by Tukey’s test at *p* < 0.05.

After oral glucose loading, serum glucose concentrations were elevated until the peak at 20–30 min, and they decreased in all groups (Figure 4A). The peak serum glucose concentrations at 30 min were much higher in the control group than the other groups, but it was observed at 20 min in the other groups. The peak was lowest in the I/R-aspirin and I/R-HCSW groups (Figure 4A). The decrease in serum glucose concentrations was much slower in the I/R-control group than in the other groups. The I/R-HCSW group showed a faster decrease in the serum glucose concentrations after the peak value than the I/R-control group (Figure 4A). According to serum glucose concentrations, insulin was released. Serum insulin concentrations were divided into two parts: After oral glucose loading, serum insulin concentrations were elevated, reached a peak at 20 min, and then decreased until 40 min, which was defined as the first part, and they were elevated again after 40 min, which was called the second part. The AUC of serum glucose and insulin concentrations were divided into two parts. During the first part of OGTT (0–40 min), the AUC of the serum glucose concentrations was higher in the control group than the Sham-control while CSW reduced the AUC as much as the Sham-control (Figure 4B). The AUC of the serum glucose concentrations during the second part of the OGTT was also highest in the I/R-control group, and it was lower in the I/R-HCSW than the Sham-control (Figure 4B). The serum insulin concentrations during OGTT were lower in the I/R-control and I/R-LCSW groups than the Sham-control groups (Figure 4C). On the other hand, the I/R-HCSW and I/R-aspirin groups showed a smaller decrease in serum insulin concentrations at 0–20 min of OGTT. The increase in the I/R-HCSW and I/R-aspirin did not reach the Sham-control.

The insulin sensitivity was determined by intraperitoneally injecting insulin (IPITT) after 6 h food deprivation. The serum glucose levels were markedly lower until 30 min and maintained or elevated during IPITT. The first part of AUC of the serum glucose concentrations was lower in the I/R-HCSW group than the I/R-control and I/R-aspirin groups, while the second part (30–90 min) of the AUC was lower in the I/R-HCSW group than the other groups (*p* < 0.05; Figure 4D).

### 3.7. Pancreatic β-Cell Mass

The individual β-cell size was larger in the I/R-control group than in the Sham-control group, and I/R-LCSW and I/R-HCSW inhibited this increase (Table 3). The individual β-cell size was similar in the I/R-HCSW and Sham-control groups. On the other hand, the total β-cell area was lower in the I/R-control group than the Sham-control group, and it decreased in the following order: Sham-control, I/R-HCSW, I/R-aspirin, I/R-LCSW, and I/R-control groups (*p* < 0.05; Table 3). The total β-cell mass calculated by multiplying the total β-cell area by the pancreas weight was lower in the I/R-control group than the Sham-control group, while CSW increased the β-cell mass as much as the Sham-control group (*p* < 0.05; Table 3). The total β-cell mass was determined by the net proliferation and apoptosis of β-cells. The β-cell proliferation was lower in the I/R-control than the Sham-control, while I/R-HCSW increased it as much as the Sham-control. Higher apoptosis of β-cells was observed in the I/R-control group than in the Sham-control group, while it decreased in the following order: I/R-LCSW, I/R-aspirin, I/R-HCSW, and Sham-control (*p* < 0.05; Table 3). 

### 3.8. Oxidative Stress and Inflammation in the Hippocampus

The differences in neurological symptoms after I/R were consistent with differences in brain cell death, possibly related to oxidative stress and inflammation in the brain. Lipid peroxide and superoxide contents, SOD activity, and proinflammatory cytokine expression were measured in the hippocampus. The lipid peroxide contents in the hippocampus were two times higher in the control group than the Sham-control, whereas they were lower in the CWS groups in a dose-dependent manner (Table 4). Superoxide contents in the hippocampus remained higher in I/R-control than Sham-control, while I/R-LCSW and I/R-aspirin reduced them compared to the I/R-control and I/R-HCSW reduced them as much as the Sham-control (Table 4). The decrease in the hippocampal superoxide contents was associated with SOD activity, which was much lower in the I/R-control than the Sham-control, and CSW and I/R-aspirin prevented the decrease in SOD activity (Table 4). TNF-α and IL-1β expression in the hippocampus was lower in the Sham-control group than in the I/R-control group, and the CSW intake partly protected the increase in artery occlusion (Table 4).

In addition to hippocampal inflammation, systemic inflammation status, measured by serum IL-1β and TNF-α concentration, also increased in I/R-controls more than Sham-control. I/R-HCSW suppressed their increase as much as the I/R-aspirin and Sham-control groups (Table 1). Thus, food intake was a primary factor in modulating the body weight gain before artery occlusion.

### 3.9. Gut Microbiome

**Figure 5:**
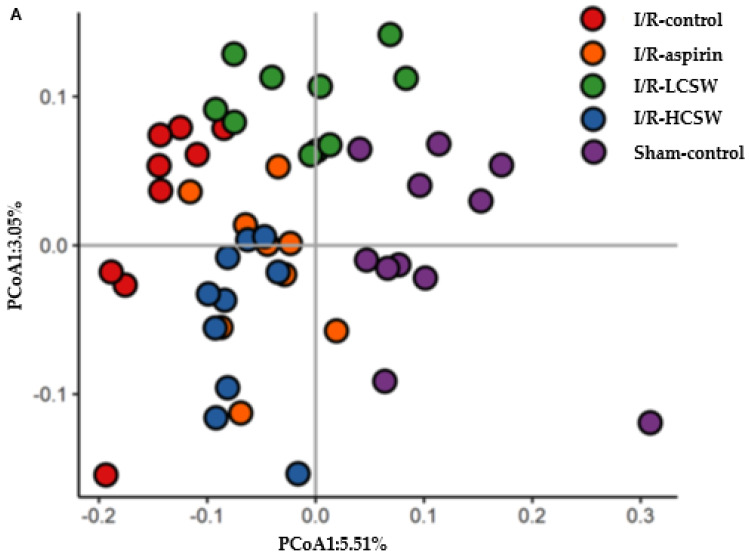

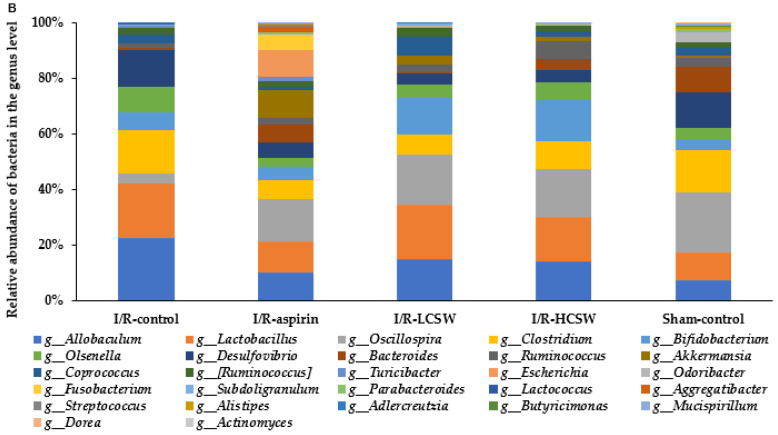
The profiles of gut microbiomes. Gerbils that underwent the transient ischemic attack by carotid artery occlusion for 8 min and reperfusion were randomly divided into four groups, as follows: (1) 0.2% cellulose (I/R-control), (2) 0.02% aspirin (I/R-aspirin), (3) 0.05% freeze-dried corn silk water extract (I/R-LCSW), and (4) 0.2% CSW (I/R-HCSW) in a high-fat diet. Sham-operated gerbils without artery occlusion had the same diet as Sham-control. At the end of the experimental period, feces from the cecum were collected, and the bacterial DNA was analyzed. The β-diversity was analyzed by the principal coordinate analysis (PCoA) of the fecal bacterial community (**A**), and the relative abundance of bacteria at the genus level (**B**) was calculated. Bars represent means ± SD (*n* = 10).

The α-diversity of the gut microbiota, as assessed by the Shannon and Chao indexes, is an indicator of the richness, dominance, and evenness of the gut bacteria, and a higher value indicates a greater richness. The Shannon and Chao indexes were lower in the I/R-control group than the Sham-control group, and they were prevented from decreasing in the I/R-LCSW and I/R-HCSW groups (Table 5). However, the I/R-aspirin group showed an improvement of α-diversity only in the Shannon index (Table 5). β-diversity is calculated as the ratio between regional and local species diversity, indicating nestedness. When samples are separated into clusters from other groups, the results indicate that the two groups have different gut microbiota. The I/R-control and Sham-control groups had significantly separated fecal bacterial groups and were also separated from the CSW and I/R-aspirin groups (Figure 5A). Only the I/R-aspirin and I/R-HCSW had similar bacteria communities.

These differences were observed in the bacteria composition. At the genus level, the relative abundance of *Lactobacillus*, *Bifidobacterium*, and *Allobaculum* was lower in the I/R-control group than the Sham-control group (Figure 5B). In contrast, it was higher in the CSW groups. The relative abundance of *Akkermansia* was higher in the I/R-aspirin and CSW groups. The relative abundance of *Clostridium*, *Desulfovibrio*, and *Oscillospira* was higher in the I/R-control group than the-Sham-control group and was suppressed by CSW intake. The *Akkermansia* proportion was higher in the I/R-aspirin and I/R-CSW groups (Figure 5B).

In metagenome analysis with Picrust2, propionate metabolism was similar in the I/R-control and Sham-control groups, while CSW and aspirin intake showed a more robust propionate metabolism (Table 5). The butyrate and starch metabolisms were much lower in I/R-control than Sham-control, while higher in I/R-CSW and I/R-aspirin, similar to Sham-control (Table 5). LPS biosynthesis was much higher in the I/R-control group than the Sham-control group, and CSW intake decreased LPS biosynthesis (Table 5).

## 4. Discussion

The present study showed that an artery occlusion for 8 min. in male gerbils induced global ischemia in the brain, while CSW intake alleviated the neuronal cell death, memory deficit, and clinical ischemic stroke symptoms. These observations are related to the reduced proinflammatory cytokines and post-stroke hyperglycemia involved in the gut microbiome changes with CSW intake. The present study is the first to demonstrate the effects of CSW on alleviating ischemic stroke and the involvement of CSW in gut microbiome alteration. CSW is rich in flavonoids and has redox activities that act as both anti- and pro-oxidant [30]. However, they mainly act as antioxidants in normal tissues to remove reactive oxygen species [31,32]. CSW has been reported to contain luteolin derivatives that can change into luteolin aglycones absorbed in detectable amounts in circulation [33]. It also includes maysin glycones, methoxymaysin, and isoorietins, known to improve oxidative stress, inflammation, and hyperglycemia [31,32].

The bioavailability of flavonoids is known to be relatively low. However, they are metabolized in the gastrointestinal tract; for example, flavonoid glycosides are changed into aglycones by hydrolase, and they are further metabolized into secondary metabolites, including benzoic, phenyl propionic, and phenylacetic acids, and hydroxy benzenes [12]. The secondary metabolites are able to be absorbed into the circulation and may protect platelet aggregation, oxidative stress, and neuroinflammation [11]. Thus, flavonoid activity is sometimes dependent on secondary metabolites produced by gut microbiota.

Ischemic stroke is partly caused by emboli initiated by activated platelets that aggregate to form clots [34], and they induce neuronal cell death by hypoxia to increase oxidative stress. It is exacerbated with hypertension, dyslipidemia, and hyperglycemia, thereby improving ischemic stroke outcomes by modulating them [35]. Platelets play a critical role in developing cardio-cerebrovascular diseases, including ischemic stroke. Abnormal platelet counts and size are biomarkers of a poor prognosis in cardiovascular diseases, and the platelet count may predict recurrent stroke and outcomes [36]. The prevention of producing and removing platelet aggregation improves blood flow to reduce the ischemic stroke risk. The prevention of post-hyperglycemia by exercise and the intake of antioxidants and chungkookjang improves blood flow and the clinical outcomes of ischemic stroke [4,5]. Platelet dysfunction in diabetic patients is related to a lack of response to the anti-aggregatory activity of nitric oxide, while acute aggressive glycemic regulation reverses with an abnormal platelet function [37]. Thus, the adverse effects of post-hyperglycemia show poorer prognostic outcomes [38].

Flavonoids have been reported to prevent platelet aggregation by several pathways as follows [39,40]: (1) inhibition of calcium mobilization to bind to calcium-dependent protein kinase by nobiletin, apigenin, genistein, quercetin, and catechin, (2) suppression of thrombin generation by quercetin and catechin, (3) blocking thromboxane receptor by apigenin, genistein, daidzein, luteolin, and quercetin, and (4) suppression of cyclooxygenase-1 activation by apigenin, genistein, quercetin, catechin, and rhoifolin. Quercetin has also been shown to regulate platelet activity by redox regulation of platelet responses to collagen via the platelet glycoprotein VI receptor, among other mechanisms [41]. However, they have not detected the parent and their metabolites in circulation [40]. CSW might alleviate platelet aggregation by mainly luteolin derivatives from luteolin glucosides, which are reported to be changed into luteolin aglycone and luteolin-3′-O-sulfate [33]. Furthermore, Applová et al. have found that the colonic flavonoid metabolite, 4-methycatechol, has greater antiplatelet activity than the commonly used cyclooxygenase inhibitor acetylsalicylic acid [12], demonstrating that the metabolites of flavonoids may be more potent than their parent compounds. The evidence supports the hypothesis that ischemic stroke might be prevented and alleviated by the actions of flavonoids and/or their metabolites. Thus, the present study suggested that the CSW intake prevented platelet aggregation potentially by luteolin and its colon metabolites by modulating intracellular calcium signaling and thromboxane receptor pathway. As a result, the gerbils with artery occlusion showed reduced blood flow and increased post-stroke hyperglycemia compared to the Sham-control group and CSW protected against ischemic stroke. Therefore, improving blood flow and glucose tolerance plays an essential role in alleviating ischemic stroke outcomes.

CSW has been reported to reduce oxidative stress and inflammation and alleviate hyperglycemia, dyslipidemia, and hypertension [31,42]. Although hypertension is more likely related to hemorrhagic stroke in younger persons, elevated arterial blood pressure is involved in the ischemic stroke risk [43]. Dyslipidemia, obesity, and hyperglycemia increase the susceptibility to blood clots by stimulating platelet aggregation. A meta-analysis of five RCT studies reported that the combination of CSW with hypertensive drugs lowers the blood pressure compared to the hypertensive drug alone (relative risk = 1.27, 1.17–1.38; *p* < 0.00001) [14]. CSW intake increased blood flow and improved glucose tolerance after artery occlusion in the present study. Furthermore, hyperglycemia was related to a decrease in β-cells after artery occlusion, and CSW prevented β-cell apoptosis in the present study. In the present study, I/R-HCSW intake lowered the serum total cholesterol and LDL cholesterol compared to the I/R-control group, but their concentrations were higher than the Sham-control group. As much as the Sham-control, the serum triglyceride concentrations were lower in the I/R-HCSW. These changes were associated with the increased hepatic gene expression of cholesterol and triglyceride synthesis in the I/R-control group compared to the I/R-HCSW and Sham-control. Previous studies showed that corn silk intake reduces serum total cholesterol concentrations and fat accumulation in the liver, decreasing mRNA expression of 3-hydroxy-3-methyl-glutaryl-coenzyme A reductase and acyl-coenzyme A: cholesterol acyltransferase in mice fed high-fat diets [44]. Furthermore, additional CSW intake with conventional medication improved dyslipidemia in patients with angina pectoris compared to conventional medication alone in a meta-analysis with four RCT studies [15]. Therefore, CSW might have beneficial activity in preventing the initiation and recurrence of ischemic stroke.

The disease status and dietary intake affect the gut microbiota composition and diversity [27,45,46]. The gut microbiome influences the host’s metabolism, primarily the glucose and lipid metabolism and inflammation. Brain diseases, including ischemic stroke, are associated with the gut microbiomes through the gut-brain axis with SCFA and proinflammatory cytokines [13,46,47]. The present study showed that stroke-induced gerbils had a lower a-diversity of the gut microbiota than the Sham-control group. CSW prevented the decreased a-diversity of the gut microbiota. The control gerbils had less beneficial bacteria than the I/R-control group, indicating that ischemic stroke might induce gut dysbiosis. CSW intake prevented gut dysbiosis by increasing *Lactobacillus*, *Bifidobacterium*, and *Akkermansia*. The gut microbiome changes were related to the CSW components that altered the starch, lipid, and protein digestion and absorption. Dietary lipid, protein, and digestible and indigestible carbohydrates influence the composition of gut bacteria. The variation in their digestion and absorption in the host modulates the gut bacteria composition to utilize the nutrients in the gut. The flavonoid components in CSW alter the bile acid secretion and digestive enzyme activity, which in turn modulates the nutrient availability for gut microbiota in the large intestines [48,49]. These changes modulate the gut bacteria composition to change SCFA, secondary bile acids, trimethylamine, and proinflammatory cytokines produced by the gut bacteria. The present study demonstrated that CSW intake increased the beneficial gut microbiota, consistent with lower serum TNF-α and IL-1β concentrations. Therefore, further studies on the direct effects of gut microbiota on ischemic stroke are needed.

Artery occlusion induces neuronal cell death by elevating oxidative stress and inflammation in the brain, leading to intestinal paralysis. Additionally, a high-fat diet aggravates intestinal paralysis and causes gut microbiome dysbiosis and intestinal barrier permeability, thereby allowing endotoxins and trimethylamine to enter the host circulation quickly. It elevates oxidative stress, proinflammatory cytokines, overactive immunity, and platelet hyperreactivity. The gut changes deliver the signals directly to the brain through the autonomous nervous system and SCFA production from the gut microbiome. Artery occlusion modulates the gut microbiome-brain axis to directly aggravate neuronal cell death by elevating oxidative stress and inflammation in the brain. It indirectly exacerbates ischemic stroke outcomes by inducing β-cell death and post-stroke hyperglycemia. We previously demonstrated that intake of chungkookjang rich in isoflavonoids and γ-PGA improves intestinal morphology, gut microbiome, glucose tolerance, and β-cell function and survival in diabetic rats [13,50]. In this study, CSW intake alleviated the clinical outcomes of ischemic stroke, which may directly reduce oxidative stress and inflammation and indirectly promote the gut microbiome-brain axis in an animal model. Serum propionate and butyrate concentrations were elevated, and beneficial bacteria in the gut microbiome were promoted and associated with reduced oxidative stress, insulin resistance, and inflammation. The benefits of CSW might be partially attributable to effects on the microbiome. The risk of stroke may increase with trimethylamine *N*-oxide produced by gut bacteria, which have detrimental effects on neurological and cardiovascular health [51]. In human stroke victims, gut dysbiosis was shown to be associated with serum inflammatory markers, interleukin-6, C-reactive protein, and white blood cell counts [52]. Even though human studies could not establish cause and effect, animal studies suggest a causal role for SCFA produced by gut bacteria in post-stroke recovery [53]. That result would be consistent with our results to demonstrate the association of serum SCFA concentrations with improved post-stroke recovery. Although this manuscript is not primarily focused on the role of gut microbiota in the prevention and recovery from ischemic stroke, the abundance of research on the topic makes it imperative to consider the possibility that gut microbiota is a factor in the data presented in the manuscript.

## 5. Conclusions

Artery occlusion produced clinical neurological symptoms, memory deficits, and reduced grip strength and blood flow compared to the Sham-control group. The post-hyperglycemia was associated with reduced β-cell mass, indicating that artery occlusion increased oxidative stress and inflammation to induce neuronal and β-cell apoptosis. These changes induced gut microbiota dysbiosis. CSW intake protected against the symptoms from artery occlusion. The protection was partially prevented via increasing blood flow, decreasing oxidative stress and inflammation, and improving gut microbiota dysbiosis. CSW intake partly prevented the decrease in beneficial bacteria, *Lactobacillus*, *Bifidobacterium*, and *Akkermansia*. CSW may have a protective effect on neuronal cell death in human stroke prevention and treatment. However, these results came from Mongolian gerbils, with similar characteristics of human stroke, but the results cannot be directly applied to humans. A further clinical study is needed.

## Figures and Tables

**Table 1:** Energy metabolism.

	I/R-Control (*n* = 10)	I/R-Aspirin (*n* = 10)	I/R-LCSW (*n* = 10)	I/R-HCSW (*n* = 10)	Sham-Control (*n* = 10)
Body weight gain during 1–3 weeks (g)	3.18 ± 0.54 ^a^	3.39 ± 0.62 ^b^	1.8 ± 0.58 ^c^	2.02 ± 0.64 ^c^	3.55 ± 0.81 ^a^
Body weight gain during 3–5 weeks (g)	−3.94 ± 0.72 ^d^	1.02 ± 0.61 ^b^	−1.20 ± 0.58 ^c^	1.05 ± 0.65 ^b^	1.85 ± 0.41 ^a^
Food intake during 1–3 weeks (g/day)	3.9 ± 0.49 ^a^	3.0 ± 0.42 ^b^	2.7 ± 0.38 ^b^	2.8 ± 0.37 ^b^	4.2 ± 0.53 ^a^
Food intake during 3–5 weeks (g/day)	2.92 ± 0.53	3.32 ± 0.57	3.14 ± 0.58	2.83 ± 0.56	3.40 ± 0.55
Corn silk water extract (mg/kg bw)	0 ^d^	4.1 ± 0.3 ^c^	19.1 ± 1.2 ^b^	73.2 ± 3.6 ^a^	0 ^d^

Gerbils that underwent transient ischemia by carotid artery occlusion for 8 min and reperfusion were randomly divided into four groups, as follows: (1) 0.2% cellulose (I/R-control), (2) 0.02% aspirin (I/R-aspirin), (3) 0.05% freeze-dried corn silk water extract (I/R-LCSW), (4) 0.2% CSW (I/R-HCSW) in a high-fat diet. Sham-operated gerbils (Sham-control) had the same diet as the I/R-control. Values are means ± SD (*n* = 10). ^a–d^ Different superscript letters of the means indicate significant differences in the means of the designated groups by Tukey’s test at *p* < 0.05.

**Table 2:** Blood flow, serum glucose and lipid profiles, and hepatic gene expression related to fat synthesis.

	I/R-Control (*n* = 10)	I/R-Aspirin (*n* = 10)	I/R-LCSW (*n* = 10)	I/R-HCSW (*n* = 10)	Sham-Control (*n* = 10)
Peak blood pressure (BPU)	22.6 ± 3.24 ^c^	23.4 ± 3.41 ^b,c^	26.4 ± 3.26 ^b^	30.4 ± 3.42 ^a^	28.7 ± 3.11 ^a^
Periods to remove the clog (min)	5.44 ± 0.75 ^a^	3.42 ± 0.43 ^c^	4.67 ± 0.55 ^b^	3.76 ± 0.58 ^c^	4.48 ± 0.57 ^b^
Serum total cholesterol (mg/dL)	157.4 ± 12.8 ^a^	153.5 ± 14.9 ^a^	150.4 ± 9.44 ^a^	135.0 ± 14.4 ^b^	121.2 ± 16.5 ^c^
Serum HDL (mg/dL)	48.6 ± 5.36	45.2 ± 3.93	44.0 ± 5.19	42.9 ± 4.85	48.2 ± 6.10
Serum LDL (mg/dL)	75.6 ± 6.81 ^a^	80.1 ± 8.60 ^a^	73.0 ± 7.00 ^a^	63.6 ± 7.13 ^b^	46.4 ± 6.26 ^c^
Serum triglyceride (mg/dL)	166.2 ± 14.0 ^a^	140.9 ± 14.8 ^b^	167.2 ± 10.7 ^a^	142.2 ± 16.0 ^b^	148.9 ± 16.5 ^b^
Serum glucose levels (mg/dL)	94.3 ± 1.68 ^a^	84.7 ± 2.17 ^b^	83.8 ± 4.04 ^b^	76.4 ± 3.28 ^c^	78.3 ± 4.08 ^c^
Serum insulin levels (ng/mL)	0.47 ± 0.07 ^b^	0.64 ± 0.09 ^a^	0.43 ± 0.05 ^b^	0.61 ± 0.09 ^a^	0.65 ± 0.10 ^a^
Hepatic expression of SREBP1c (AU)	1.0 ± 0.0 ^a^	0.98 ± 0.11 ^a^	0.94 ± 0.10 ^a^	0.84 ± 0.09 ^b^	0.81 ± 0.09 ^b^
Hepatic expression of FAS (AU)	1.0 ± 0.0 ^a^	0.78 ± 0.09 ^b^	0.98 ± 0.11 ^a^	0.83 ± 0.09 ^b^	0.85 ± 0.09 ^b^

Gerbils that underwent the transient ischemic attack by carotid artery occlusion for 8 min and reperfusion were randomly divided into four groups, as follows: (1) 0.2% cellulose (I/R-control), (2) 0.02% aspirin (I/R-aspirin), (3) 0.05% freeze-dried corn silk water extract (I/R-LCSW), and (4) 0.2% CSW (I/R-HCSW) in a high-fat diet. Sham-operated gerbils (Sham-control) had the same diet as the I/R-control. BPU, blood pressure unit; HDL, high-density lipoprotein; LDL, low-density lipoprotein; FAS, fatty acid synthase; SREBP1c, sterol regulatory element-binding proteins. Values are means ± SD (*n* = 10). ^a–c^ Different superscript letters of the means indicated significant differences in the means of the designated groups by Tukey’s test at *p* < 0.05.

**Table 3:** Pancreatic β-cell mass.

Variables	I/R-Control (*n* = 10)	I/R-Aspirin (*n* = 10)	I/R-LCSW (*n* = 10)	I/R-HCSW (*n* = 10)	Sham-Control (*n* = 10)
Individual β-cell size (μm^2^)	7.97 ± 0.98 ^a^	6.76 ± 0.70 ^b^	6.94 ± 0.77 ^b^	6.28 ± 0.73 ^b,c^	6.05 ± 0.71 ^c^
β-cell area (%)	20.3 ± 3.15 ^c^	24.9 ± 3.34 ^b^	23.9 ± 3.04 ^b^	27.7 ± 3.46 ^a^	27.9 ± 3.28 ^a^
Total β-cell mass (mg)	0.94 ± 0.12 ^c^	1.12 ± 0.14 ^b^	1.13 ± 0.14 ^b^	1.28 ± 0.15 ^a^	1.24 ± 0.14 ^a^
BrdU^+^ cells (% BrdU^+^ cells of islets)	5.11 ± 0.59 ^b^	5.61 ± 0.66 ^a,b^	5.58 ± 0.71 ^a,b^	6.11 ± 0.67 ^a^	5.97 ± 0.65 ^a^
Apoptosis (% apoptotic bodies of islets)	22.1 ± 2.54 ^a^	16.2 ± 2.02 ^c^	18.5 ± 2.14 ^b^	15.7 ± 1.74 ^c^	14.6 ± 1.59 ^c^

Gerbils that underwent the transient ischemic event by carotid artery occlusion for 8 min and reperfusion were randomly divided into four groups, as follows: (1) 0.2% cellulose (I/R-control), (2) 0.02% aspirin (I/R-aspirin), (3) 0.05% freeze-dried corn silk water extract (I/R-LCSW), and (4) 0.2% CSW (I/R-HCSW) in a high-fat diet. Sham-operated gerbils (Sham-control) had the same diet as the I/R-control. Values are means ± SD (*n* = 10). ^a–c^ Different superscript letters of the means indicated significant differences in the means of the designated groups by Tukey’s test at *p* < 0.05.

**Table 4:** Oxidative stress and inflammation markers.

	I/R-Control (*n* = 10)	I/R-Aspirin (*n* = 10)	I/R-LCSW (*n* = 10)	I/R-HCSW (*n* = 10)	Sham-Control (*n* = 10)
Hippocampal lipid peroxides (MDA μmol/g tissue)	0.43 ± 0.06 ^a^	0.30 ± 0.05 ^b^	0.33 ± 0.05 ^b^	0.25 ± 0.04 ^c^	0.21 ± 0.04 ^c^
Superoxide contents in the hippocampus (μM NBT/mg protein)	6.83 *±* 0.49 ^a^	5.85 ± 0.52 ^b^	5.91 *±* 0.46 ^b^	5.14 *±* 0.43 ^c^	5.02 *±* 0.41 ^c^
SOD in the hippocampus (U/mg protein)	503 *±* 44 ^a^	581 *±* 39 ^b^	573 *±* 42 ^b^	625 *±* 45 ^c^	638 *±* 42 ^c^
Relative mRNA expression of hippocampal TNF-α (AU)	1.0 ± 0 ^a^	0.71 ± 0.09 ^b,c^	0.79 ± 0.09 ^b^	0.66 ± 0.08 ^c^	0.60 ± 0.07 ^c^
Relative mRNA expression of hippocampal IL-1β (AU)	1.0 ± 0 ^a^	0.76 ± 0.08 ^b^	0.78 ± 0.09 ^b^	0.72 ± 0.09 ^b,c^	0.66 ± 0.08 ^c^
Serum interleukin-1β levels (pg/mL)	10.7 ± 1.17 ^a^	7.84 ± 0.88 ^c^	8.78 ± 0.91 ^b^	7.94 ± 0.84 ^c^	7.76 ± 0.79 ^c^
Serum TNF-α levels (pg/mL)	24.8 ± 2.94 ^a^	18.4 ± 2.17 ^c^	21.4 ± 2.51 ^b^	18.9 ± 2.14 ^c^	17.5 ± 1.94 ^c^

Gerbils that underwent the ischemic stroke by carotid artery occlusion for 8 min and reperfusion were randomly divided into four groups, as follows: (1) 0.2% cellulose (I/R-control), (2) 0.02% aspirin (I/R-aspirin), (3) 0.05% freeze-dried corn silk water extract (I/R-LCSW), and (4) 0.2% CSW (I/R-HCSW) in a high-fat diet. Sham-operated gerbils (Sham-control) had the same diet as the I/R-control. MDA, malondialdehyde; NBT, nitroblue tetrazolium solution; SOD, superoxide dismutase; TNF-α, tumor necrosis-α; IL-1β, interleukin-1β; AU, arbitrary unit. Values are means ± SD (*n* = 10). ^a–c^ Different superscript letters of the means indicated significant differences in the means of the designated groups by Tukey’s test at *p* < 0.05.

**Table 5:** Serum short-chain fatty acid (SCFA) concentrations, α-diversity, and metagenome analysis.

	I/R-Control (*n* = 10)	I/R-Aspirin (*n* = 10)	I/R-LCSW (*n* = 10)	I/R-HCSW (*n* = 10)	Sham-Control (*n* = 10)
SCFA by gas chromatography					
Serum acetate	0.782 ± 0.003 ^b^	0.837 ± 0.011 ^b^	1.036 ± 0.003 ^a^	0.958 ± 0.006 ^a^	0.838 ± 0.004 ^b^
Serum propionate	0.403 ± 0.003 ^b^	0.411 ± 0.011 ^a,b^	0.404 ± 0.003 ^b^	0.414 ± 0.006 ^a^	0.408 ± 0.004 ^a,b^
Serum butyrate	0.368 ± 0.003 ^b^	0.368 ± 0.011 ^b^	0.375 ± 0.003 ^b^	0.384 ± 0.006 ^a^	0.370 ± 0.004 ^b^
α-diversity					
Shannon index	5.0 ± 0.4 ^c^	5.6 ± 0.6 ^b^	5.4 ± 0.8 ^b,c^	5.8 ± 0.5 ^b^	6.1 ± 0.6 ^a^
Chao index	9763 ± 924 ^b^	9562 ± 849 ^b^	11,467 ± 1045 ^a^	12,042 ± 1175 ^a^	12,093 ± 1345 ^a^
Metagenome analysis by Picrust2					
Propionate metabolism	1.05 ± 0.09 ^b^	1.14 ± 0.04 ^a^	1.09 ± 0.07 ^a,b^	1.07 ± 0.07 ^b^	1.08 ± 0.06 ^b^
Butanoate metabolism	0.96 ± 0.07 ^b^	1.16 ± 0.10 ^a^	1.11 ± 0.14 ^a^	1.12 ± 0.06 ^a^	1.14 ± 0.11 ^a^
LPS biosynthesis	0.29 ± 0.04 ^a^	0.26 ± 0.12 ^a^	0.18 ± 0.06 ^b^	0.11 ± 0.08 ^b^	0.12 ± 0.09 ^b^
Starch metabolism	1.75 ± 0.16 ^c^	2.28 ± 0.04 ^b^	2.35 ± 0.45 ^a,b^	2.30 ± 0.05 ^b^	2.76 ± 0.11 ^a^

Gerbils that underwent the transient ischemic attack by carotid artery occlusion for 8 min and reperfusion were randomly divided into four groups, as follows: (1) 0.2% cellulose (I/R-control), (2) 0.02% aspirin (I/R-aspirin), (3) 0.05% freeze-dried corn silk water extract (I/R-LCSW), and (4) 0.2% CSW (I/R-HCSW) in a high-fat diet. Sham-operated gerbils (Sham-control) had the same diet as the I/R-control. LPS, lipopolysaccharide. Values are means ± SD (*n* = 10). ^a–c^ Different superscript letters of the means indicated significant differences in the means of the designated groups by Tukey’s test at *p* < 0.05.

## Data Availability

Data is contained within the article.

## Supplementary Materials

The following supporting information can be downloaded at: www.mdpi.com/article/10.3390/antiox11010168/s1, Figure S1. Hippocampal cell death after ischemic stroke.
