# Supplementary material for: Protection against Neurological Symptoms by Consuming Corn Silk Water Extract in Artery-Occluded Gerbils with Reducing Oxidative Stress, Inflammation, and Post-Stroke Hyperglycemia through the Gut-Brain Axis
Source: Antioxidants (Basel). 2022 Jan 16;11(1):168. doi: 10.3390/antiox11010168 (PMC8773031; doi:10.3390/antiox11010168)
Supplement: Supplementary file 1 [file antioxidants-11-00168-s001.zip › antioxidants-1455608-supplementary.pdf]

Supplementary Figure S1. Hippocampal cell death after ischemic stroke.

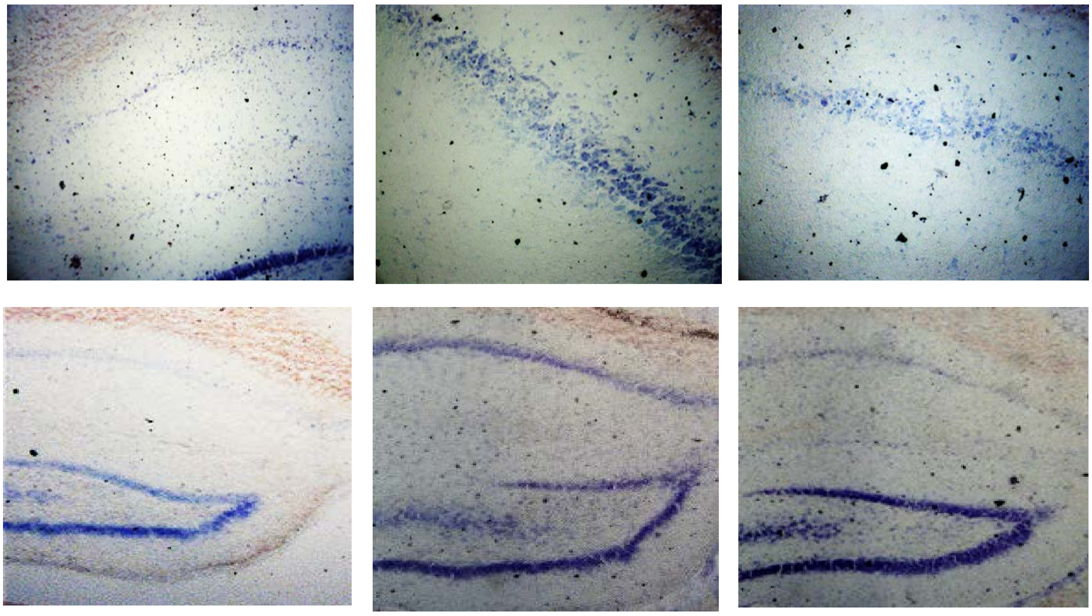

I/R-control **I/R-aspirin** **I/R-LCSW**

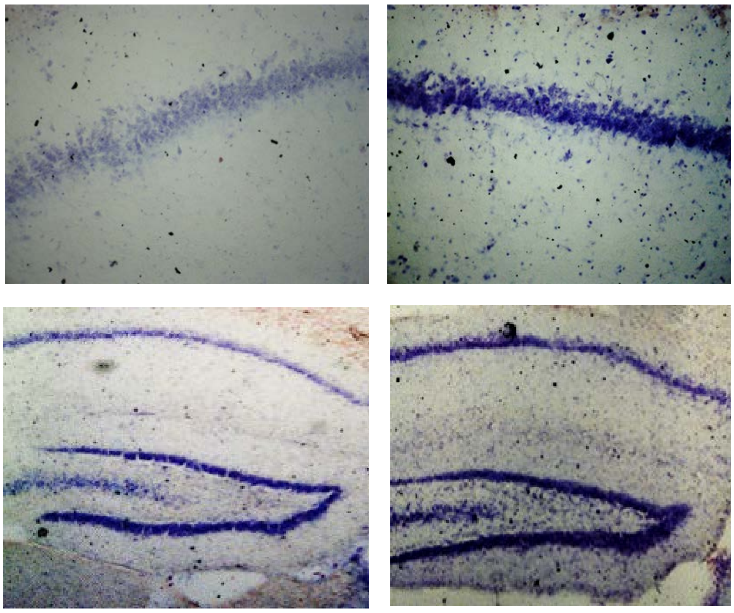

I/R-HCSW **Sham-control**

Gerbils that underwent the ischemic by carotid artery occlusion for 8 mins were randomly divided into 4 groups, as follows: : 1) 0.2% cellulose (I/R-control), 2) 0.02% aspirin (I/R-aspirin), 3) 0.05% freeze-dried corn silk water extract (I/R-LCSW), 4) 0.2% CSW (I/R-HCSW) in in a high-fat diet. Sham-operated gerbils without artery occlusion had the same diet as Sham-control. At the end of the experiment, the brain section had cresyl violet staining in the hippocampus (A). Blue staining cells indicated live cells. Above picture magnification X50, below magnification X200
